# Analysis of consumer food purchase data used for outbreak investigations, a review

**DOI:** 10.2807/1560-7917.ES.2018.23.24.1700503

**Published:** 2018-06-14

**Authors:** Frederik T Møller, Kåre Mølbak, Steen Ethelberg

**Affiliations:** 1 Department of Infectious Disease Epidemiology and Prevention, Statens Serum Institut, Copenhagen, Denmark; 2 Department of Epidemiology Research, Statens Serum Institut, Copenhagen, Denmark; 3 Institute of Veterinary and Animal Sciences, University of Copenhagen, Denmark

**Keywords:** food-borne infections, outbreaks, epidemiology, zoonotic infections, salmonella, review

## Abstract

Investigations of food-borne outbreaks are frequently unsuccessful and new investigation methods should be welcomed. **Aim:** Describe the use of consumer purchase datasets in outbreak investigations and consider methodological and practical difficulties. **Methods:** We reviewed published papers describing the use of consumer purchase datasets, where electronic data on the foods that case-patients had purchased before onset of symptoms were obtained and analysed as part of outbreak investigations. **Results:** For the period 2006–17, scientific articles were found describing 20 outbreak investigations. Most outbreaks involved salmonella or Shiga toxin-producing Escherichia coli and were performed in eight different countries. The consumer purchase datasets were most frequently used to generate hypotheses about the outbreak vehicle where case-interviews had not been fruitful. Secondly, they were used to aid trace-back investigation, where a vehicle was already suspected. A number of methodological as well as (in some countries) legal and practical impediments exist. **Conclusions:** Several of the outbreaks were unlikely to have been solved without the use of consumer purchase datasets. The method is potentially powerful and with future improved access to big data purchase information, may become a widely applicable tool for outbreak investigations, enabling investigators to quickly find hypotheses and at the same time estimate odds ratios or relative risks hereof. We suggest using the term ‘consumer purchase data’ to refer to the approach in the future.

## Introduction

Food-borne illnesses are a considerable cause of mortality, in particular among children, in the developing world and an important cause of morbidity in the developed world. Work from the World Health Organization (WHO) Food-borne Disease Burden Epidemiology Reference Group has estimated that 600 million food-borne illnesses occurred worldwide in the year 2010, leading to 420,000 deaths. In the WHO European Region, an estimated annual 23 million illnesses occur [1]. In the United States (US), it has been estimated that food-borne illness that can be specifically attributed to the major pathogens affects more than 48 million citizens annually [2] and amounts to an economic burden of several billion US dollars [3].

In the European Union (EU), food-borne disease outbreaks occur also frequently. In 2015, 4,362 outbreaks were of such relevance that they were reported to the European Centre for Disease Prevention and Control (ECDC) and the European Food Safety Authority (EFSA) [4], and the control of outbreaks lies at the heart of the effort to reduce food-borne illnesses. Investigations of outbreaks help stop disease transmission, contribute to our understanding of the underlying outbreak drivers, and help to improve food safety. However, investigating food-borne outbreaks is often not a straightforward task. For dispersed outbreaks where microbiological proof often cannot readily be obtained, the steps of finding hypotheses, generally done via extensive interviews with outbreak cases – and proving/disproving hypotheses, generally done by use of analytical epidemiology, are difficult but critical factors for the success of the investigation. Outbreaks caused by agents with a long incubation time or by several different products, products with long shelf lives, low brand recognition, or representing subsets of foods that are very commonly consumed are especially hard to resolve through patient interviews. Thus alternative methods for their investigation should be considered. One such method utilises individualised consumer purchase data to resolve outbreaks, taking advantage of the fact that many retailers collect and store this information in searchable databases. The method has been used irregularly over the past decade with heterogeneous reporting and methodology and more wide-scale, systematic implementation has not ensued.

In this article, we review the literature on consumer purchase data use in outbreak investigations. We classify different categories of usage in the literature and address methodological difficulties and further outline some future perspectives.

## Methods

We searched for and included published studies in English involving food-borne outbreaks where consumer purchase data (e.g. loyalty card or credit/debit card data) were applied in outbreak investigations. The search was conducted in August 2016 using the PubMed database and Google Scholar and was repeated in October 2017 with the additional inclusion of the Scopus and Web of Science databases. The latter search combined the search terms (‘disease outbreak*’ AND ‘food*’) OR ‘food contamination*’ OR ‘foodborne disease’ with the search terms ‘card*’ OR ‘receipt*’ OR ‘loyalty* OR ‘till*’ or ‘membership*’. MeSH terms were used in Medline. In addition, further studies cited within the papers or already known to the author group or collaborators were also included. The search included papers published from January 2006 to 20 October 2017. Papers describing simulated outbreaks were excluded, as were papers where consumer purchase data were not applied in relation to food-borne outbreak investigations. The search was done by one author. Papers selected for narrative synthesis were assessed by two authors and discussed within the author group to reach consensus on methodology. A classification of use was made within the categories: hypothesis generation, trace back, corroboration of hypothesis, analytical usage.

References in papers found through the above-described search showed that, in each study, only a few other studies using the consumer card method were cited, possibly due to a marked heterogeneity in the nomenclature regarding the use of consumer purchase data. The terms used in the found studies [5-24] included: household shopping receipts [18], consumer loyalty cards [8], shopper cards data [23], customer loyalty cards [10], supermarket loyalty cards [21], warehouse store membership card [15], loyalty card [20], grocery store loyalty card [5], credit card information [24], shopper-card information [13,14,18] and till receipts [11].

In this paper we have used the term ‘consumer purchase data’ to cover different sources of information for purchases of food, e.g. a credit card or a loyalty card, to cover the entire process of using the data as a method for outbreak investigations.

## Results

The results of the publication search strategy are shown in the Figure. Over the study period, 20 papers published in international peer-reviewed journals were identified describing outbreaks where consumer purchase data were collected and used for food-borne outbreak investigations [5-24].

**Figure:**
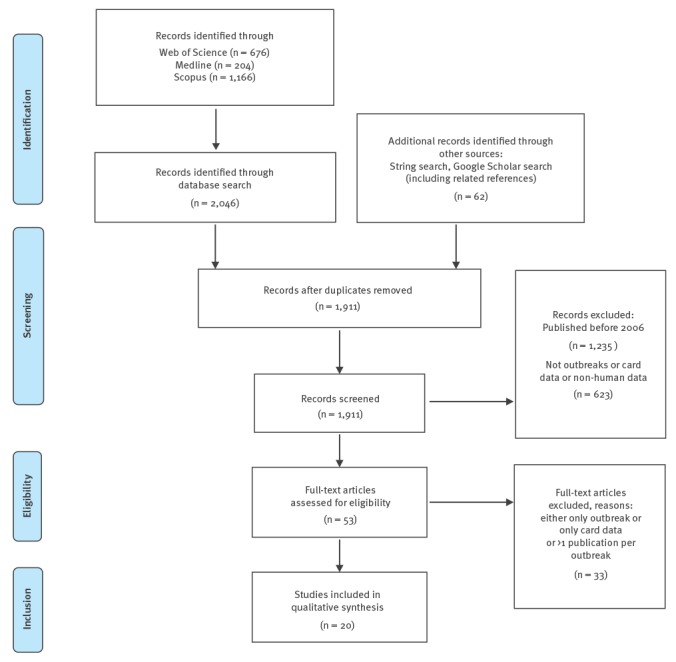
Flowchart of publication search results, 20 October 2017

### Outbreak characteristics

Table 1 gives an overview of the outbreaks. The outbreaks were primarily caused by *Salmonella enterica* of different serotypes and subtypes (12 outbreaks) [10-21], followed by Shiga-toxin producing *Escherichia coli* (STEC) of different O-groups (3 outbreaks) [22-24], hepatitis A virus (3 outbreaks) [6-8], as well as *Listeria monocytogenes* [5] and *Cyclospora cayetanensis* [9] (1 outbreak each). The outbreaks took place in North America [5,7,8,10,12-17] or Europe [6,9,11,18-24] and, except for two, were dispersed outbreaks, extended over time and geographical area. They were mostly nationwide outbreaks, although two outbreaks were international. Two outbreaks were point-source outbreaks examined using a cohort set-up [11,22]. The outbreaks were of varying size, the number of outbreak cases ranged from six to more than 1,000, with a median of 63 cases. The duration of the outbreaks ranged from 1 week to more than 1 year. The outbreak source was identified, with varying degrees of supporting evidence presented, for all but one [19] of the 20 investigated outbreaks.

**Table 1:** Overview of published papers of food-borne outbreak investigations using consumer purchase data (CPD) for investigations, 1 January 2006−20 October 2017

Agent causing outbreak	No of cases	Country	Source or vehicle	Duration (weeks)	Year of outbreak	Reference and year of publication
*Cyclospora cayetanensis*	29	Canada	Organic basil	13	2007	[5] Shah et al., 2009
**Hepatitis A virus**						
Hepatitis A virus	103	Denmark, Sweden, Norway, Finland	Frozen strawberries produced in Belgium	35	2013	[6] Gillesberg Lassen et al., 2013
Hepatitis A virus	165	United States	Frozen pomegranate arils produced in Turkey	19	2013	[7] Collier et al., 2014
Hepatitis A virus	9	Canada	Frozen fruit blend	10	2012	[8] Swinkels et al., 2014
*Listeria monocytogenes*	6	Switzerland	Cooked ham	14	2011	[9] Hächler et al., 2013
***Salmonella *spp**						
*Salmonella* Chester	33	Canada	Pork product	9	2010	[10] Taylor et al., 2012
*S.* Enteritidis	66	United Kingdom (London)	Rotisserie chicken	1	2009	[11] Zenner et al., 2014
*S.* Enteritidis	43	United States (5 states)	Pine nuts produced in Turkey	12	2011	[12] Bedard et al., 2014
*S.* Heidelberg	134	United States (13 states)	Chicken meat	46	2012	[13] Grinnell et al., 2013
*S.* Heidelberg	136	United States (34 states)	Ground turkey	37	2011	[14] Routh, et al., 2015
*S.* Montevideo and *S.* Senftenberg	283	United States (44 states)	Pepper (spice), produced in Asia	41	2009	[15] Gieraltowski et al., 2012
*S.* Newport	42	United States	Ground beef	10	2007	[16] Schneider et al., 2011
*S.* Newport	6	United States (1 state)	Fresh blueberries	3	2010	[17] Miller et al., 2013
*S.* Strathcona	71	Denmark (plus Germany, Italy, Austria, Belgium)	Tomatoes produced in Italy	23	2011	[18] Müller et al., 2016
*S.* Typhimurium	1,054	Denmark	Unknown	29	2008	[19] Ethelberg et al., 2008
*S.* Typhimurium, monophasic	110	France	Dried pork sausage	18	2010	[20] Bone et al., 2010
*S.* Typhimurium, monophasic	337	France	Dried pork sausage	7	2011	[21] Gossner et al., 2012
**Shiga toxin-producing *Escherichia coli***						
STEC O104	60	Germany	Sprouts	9	2011	[22] Wilking et al., 2012
STEC O157	15	France	Beef burgers	6	2012	[23] Barret et al., 2013
STEC O26	20	Denmark	Organic beef salami	9	2007	[24] Ethelberg et al., 2009

STEC: Shiga toxin-producing *Escherichia coli.*

Table 2 lists further details about each publication, categorising purchase data source and the general use of the method into categories. For the dispersed outbreaks, the purchase data were accessed from supermarket or similar type of store databases. The databases holding the cash register information were searched for specific purchase transactions. These were based on the case/consumer loyalty card number, credit/debit card number or simply the amount paid coupled with date and branch of store, information that was derived from cases’ (web) bank statements following purchases with payment cards. Consumer purchase data were used for two major purposes: source hypothesis-generation [5,9-12,18-21,24] and food trace-back investigations [5-8,10,13-17,20,21,23]. A few papers described further types of application of the methodology [7,11,21,22]. Below, the use of consumer purchase data are described in more detail including examples.

**Table 2:** Category of use of consumer purchase data (CPD) for food-borne outbreak investigations, 1 January 2006−20 October 2017

Reference	Purchase data source	Category of use of CPD	Description of use of CPD	Type of outbreak	Type of vehicle
[5] Shah et al., 2009	Loyalty card; 8 cases.	Hypothesis generation and trace-back investigation	CPD used for hypothesis generation/ support of hypothesis and aid in trace back	Dispersed one-province cyclosporiasis outbreak	Organic basil was the most likely vehicle
[6] Gillesberg Lassen et al., 2013	Debit card information, several supermarkets; no. of cases not stated (< 10).	Trace back	Vehicle (frozen berries) found by case–control study; CPD used to identify type and identity of product.	National, later international (4 countries) hepatitis A outbreak	Brand of frozen strawberries sold in (internationally operating) supermarket chain
[7] Collier et al., 2014	Data from membership/loyalty cards from a retailer; no. of cases not stated.	Case finding, trace back, targeted intervention of exposed (information, post-exposure vaccination)	CPD Improved validity of initial hypothesis and targeted post exposure prophylaxis with both hepatitis A virus vaccine and immunoglobulin.	Dispersed national hepatitis A outbreak	Frozen pomegranate arils
[8] Swinkels et al., 2014	Loyalty card purchases in 3-month period; 6 cases.	Trace back	Vehicle identified in part using classical epidemiology, CPD used to locate particular producer and confirm the source. No case–control study done.	Dispersed province-wide hepatitis A outbreak	Frozen berry blend
[9] Hächler et al., 2013	Shopper cards/loyalty cards; 4 cases.	CPD support existing evidence	Supported existing evidence, use delayed by legal clarification. Consent from the patients and the retail company.	Dispersed local listeria outbreak	Cooked ham
[10] Taylor et al., 2012	Loyalty card purchases; 4 cases.	Assists hypothesis generation, trace back	Epidemiological investigation points to vehicle. CPD in subset of cases corroborates and leads to fast trace back.	Dispersed multi-province salmonella outbreak	Ready-to-eat pork product, known as head cheese
[11] Zenner et al., 2014	Till entries and receipts from single restaurant; 41 cases.	Hypothesis generation, analytical study	Helps locate dish on menu in take-away restaurant + makes analytical argument by comparing sale over different time periods.	Point-source (geographical) outbreak associated with single restaurant	Chicken dish, one item of many on a restaurant menu
[12] Bedard et al., 2014	Shopper card purchases, no of cases not stated (< 10)	Hypothesis generation	CPD gives 3 distinct hypotheses, leads to source identification by microbiological testing.	Local county investigation and multi-state cluster	Pine nuts sold in supermarket/stores
[13] Grinnell et al., 2013	Shopper card purchases; 9 cases.	Trace back	Standard epidemiological methods identify vehicle, CPD used to zoom in on producer and exact product.	Dispersed multi-state salmonella outbreak	Industrial chicken products sold in supermarket chain(s)
[14] Routh et al., 2015	Loyalty card purchases; 3 cases.	Trace back	Trace back (helping to identify the vehicle, combined with traditional methods).	Dispersed national salmonella outbreak	Ground turkey
[15] Gieraltowski et al., 2012	Store membership card purchases; 7 cases initially, 19 cases at late stage.	Hypothesis generation (and trace back)	CPD information points to specific hypothesis. Also strongly aids trace back.	Dispersed multi-state salmonella outbreak, 2 serotypes and several vehicles	Salamis made with contaminated black and red pepper (dried spices)
[16] Schneider et al., 2011	Loyalty cards; 11 cases.	Aided trace back	CPD improves validity of questionnaire findings. CPD used to target trace back combined with records of beef processing.	National, multistate salmonella outbreak	Ground beef
[17] Miller et al., 2013	Shopper card purchases; 3 cases.	Trace back	Vehicle suspected by epidemiological methods, small outbreak, evidence in-conclusive. CPD gives GTIN numbers which leads to precise trace back, identifying product.	Dispersed, but small, salmonella outbreak in part of 1 state	Fresh berries, sold in supermarket chain, traced back to specific producer
[18] Müller et al., 2016	Digital receipts from cashier systems from 2 supermarket chains of purchases in 6-week period; 15 cases.	Hypothesis generation	Initial hypothesis-generating interviews are inconclusive, but points to 2 supermarkets. CPD leads to quite specific hypothesis. Followed by traditional case–control study.	Dispersed national salmonella outbreak	Particular type of tomatoes, hidden among all tomatoes in interviews
[19] Ethelberg et al., 2008	Debit cards; digital receipts from several supermarket chains purchases in 6 week period; no. of cases not stated (ca 25).	Hypothesis generation	Many different investigation methods in use. CPD applied on several supermarkets/shops. No common pattern was identified.	Large and prolonged nation-wide salmonella outbreak	Vehicle/source never identified
[20] Bone et al., 2010	Loyalty card purchases three weeks before onset; 9 cases.	Trace back and corroboration of hypothesis	Epidemiological investigation points to vehicle. CPD corroborates (9/9 cases bought product) and points to single brand. Recall without case–control study or microbiological proof.	Dispersed national salmonella outbreak	Dried salami, distributed nation-wide, sold in single supermarket chain
[21] Gossner et al., 2012	Loyalty card purchases; 39 cases.	Trace back and semi-analytical use	Epidemiological investigation points to vehicle. Focused CPD corroborates and points to single brand. Proportions used for likelihood argument. Recall without case–control study or microbiological proof.	Dispersed national salmonella outbreak	Dried salami, distributed nation-wide, sold in supermarket chain
[22] Wilking et al., 2012	Employee cards used for cafeteria sales; 23 cases and 30 controls.	Analytical study	CPD data used for nested case–control study within cohort of company workers	Point-source outbreak, sub-outbreak within large national STEC outbreak	Raw sprouts served as part of lunch meals
[23] Barret et al., 2013	Shopper card purchases; 5 cases (though not clearly stated).	Trace back	Find the exact brand of product after vehicle has been identified using epidemiological methods	Regional (sub-national) STEC outbreak	Fresh ground beef (burgers) sold in supermarket chain.
[24] Ethelberg et al., 2009	Debit cards; digital receipts from purchases from 2 supermarket chains in 6-week period; 7 cases.	Hypothesis generation.	Initial hypothesis-generating interviews are inconclusive, but points to single supermarket. CPD leads to specific hypothesis. Further proof from case–control study and microbiological testing.	Dispersed national STEC outbreak among children	Organic, fermented salami made of beef, distributed nation-wide, sold in single supermarket chain

CPD: consumer purchase data; GTIN: global trade item number; STEC: Shiga toxin-producing *Escherichia coli*.

### Hypothesis generation

In all reports of dispersed outbreaks, the investigators followed the standard approach of aiming to generate a hypothesis as to the vehicle of the outbreak, using hypothesis-generating interviews with standardised questionnaires. Consumer purchase data were used in situations where the initial hypothesis-generating activities did not lead to a hypothesis or where the product category suggested was unspecific. Case-patients gave permission to the outbreak investigation teams to access sources of information that could be used to perform a search. This involved loyalty or ‘shopper’ card numbers, credit or debit card numbers or (online) bank statements detailing supermarket purchases. This information was then taken to the retailers to search for computerised data on all specific products bought during each particular transaction done before onset of symptoms. The time window of purchases was defined as 3 weeks [18,20], 6 weeks [24] or 3 months [8] before onset of symptoms or was not mentioned. Some investigators performed the search manually, others in a semi-automated manner by retrieving the data from a central supermarket computer system.

In seven studies (Table 2), the use of the method gave a narrower range of candidate products (often only one) than the range of products initially identified using hypothesis-generating interviews [5,10,12,15,18,20,24]. In one outbreak, no hypotheses were found [19]. The number of cases from whom consumer purchase information was obtained ranged from four to 43 in the reviewed studies. Following use of the method, testing of hypotheses was generally performed using standard methods, such as case–control studies or microbiological analysis of foods.

In one example, a local cluster of *Salmonella* Enteritidis cases detected by routine surveillance was investigated. Use of the methods on a subset of cases identified three possible hypotheses, tomatoes, avocado and pine nuts. Further investigation, including microbiological examination of products collected from cases’ homes identified pine nuts as the source of the outbreak [12]. In a second example of STEC O26 infections affecting primarily children under the age of 3 years, interviews with parents failed to produce workable hypotheses. Comparison of purchase data from seven families revealed that six of these had bought a specific brand of organic beef salami before onset, a product that none of the parents had reported during the interviews. A subsequent case–control study corroborated this product as the source of infections and the outbreak strain was later also isolated from the product [24].

### Analytical epidemiology

None of the studies of dispersed outbreaks used consumer purchase data for a regular analytical study, i.e. to produce a measure of association, such as an odds ratio. However, in several instances, the results obtained from use of the method were of sufficient specificity to produce convincing evidence as to the outbreak source. In an outbreak of salmonellosis in France, epidemiological investigations led to the hypothesis that salami-style pork sausage was the vehicle. Of 39 cases whose shopping data in one supermarket chain were retrieved, 22 had bought such sausages and 15 had bought exactly the same product from a single producer. Using overall sales data from the supermarket chain, this product was found to constitute only 3% of all salami sales. Based on this, a recall of the sausage was undertaken [21].

Two reports concerned analytical usage in a point-source outbreak setting. Following an *S*. Enteritidis outbreak found to be associated with a take-away restaurant in London, sales data were used to point to a particular chicken meal. This was done by comparing sales made by cases with sales made by other costumers at the same hour the day before [11]. The second report concerned an outbreak within the outbreak of the larger German O104 STEC outbreak in 2011 [25]. It occurred among employees of a company and was linked to the company canteen where employees paid for lunch meals using their employee access cards. This meant that the employees’ lunch choices were being electronically registered. This way, in a retrospective nested case–control study within the cohort, the strength of an association between cases and sprout-containing salad meals could be estimated [22].

### Trace back or trace forward

In 13 studies, trace-back and/or trace-forward investigation was performed by use of consumer purchase data, once a probable source of the infections had been identified (Table 2) [5-8,10,13-17,20,21,23]. The source of the infections in the studies ranged from vegetables, fruits and nuts (raw tomatoes, organic basil, blueberries, frozen fruit blend, pine nuts), to meat products (including beef burgers, poultry, delicatessen sausages and meat as well as ground turkey, dried pork sausages, fermented sausage, and rotisserie chicken (Table 1). In some studies, this trace back formed part of the evidence for what constituted the source of the outbreak.

In one outbreak, hypothesis generation was guided by loyalty card-derived purchase data, which revealed a specific type of salami as a common food purchase. The purchase data therefore also facilitated locating the distributor. The resulting trace-back investigation indicated that dried pepper, used as an ingredient in the salamis, was the probable source of the outbreak. Trace forward led to further identification of tainted products including human cases affected by a second *Salmonella* serotype found in a red pepper storage facility, thereby extending the understanding of the outbreak [15]. In hepatitis A virus outbreaks in Canada and Scandinavia, frozen fruit/berries were identified as sources. The long incubation period and the fact that multiple similar product categories existed made trace back a challenge. Analysis of purchase data records allowed investigators to pinpoint the precise products via the food product identification codes without which trace back would most likely not have been possible [6,8,17].

Finally, in one outbreak [7] consumer purchase data was used to directly target exposed individuals. In this hepatitis A virus outbreak in the US, purchase data was used to define cases (purchase/exposure being part of the case definition) and further to warn customers who had purchased the product by use of automated voice-message phone calls and to target post-exposure immunisation to exposed costumers. This was carried out by the affected retail chain, and not through data sharing with public health officials.

## Discussion

In this review, we found that consumer purchase data have been applied successfully in several phases of outbreak investigations. In the studies reviewed, the method was used for forming or assisting in forming hypotheses for the source/vehicle of the outbreaks where prior interviews had proven insufficient. Additionally, purchase data often aided source finding, providing a product subtype and sometimes even a lot or batch number. In some outbreaks, time to product recall was reduced, in others it was unlikely that the source would have been found, had it not been for the purchase data. The low number of documented purchase events needed in many of the studies to identify a probable source is a promising finding. Conversely, 20 papers published over the last decade represents a rather low number, suggesting the existence of obstacles to widespread use. We suggest using the term ‘consumer purchase data’ in future to refer to the approach as we think this term better captures the different aspects of the approach that we encountered than terms using the word ‘card’.

Critical steps in the investigation of food-borne outbreaks concern identification of suspect food products and providing proof of the source beyond reasonable doubt. We believe the evidence available from the papers reviewed here suggests that the use of purchase data may be a generalisable investigation method that could be very attractive for the investigation of challenging food-borne outbreaks. As some of the papers showed, searching through datasets across households with case-patients for common purchases may often be a more powerful method than the standard methods of interviewing case-patients, which are subject to incomplete recall. Interviews are less efficient in situations where, for instance, the period between interview and exposure is long [26] or the food is of a kind that is unlikely to be reported on, such as foods that are hard to remember (e.g. sprouts), food ingredients or sub-batches of common foods.

Establishing proof is generally possible using one of three strategies: microbiological evidence (finding the disease agent in the food using a specific typing method), epidemiological evidence (showing that a strong association between case status and a specific food consumption is present) or food supply evidence (showing a correlation between cases exposure and the presence of the incriminated foods). The papers we found generally did not use the purchase data method with the purpose of establishing proof. Potentially, however, strong evidence could be established by use of the purchase data method. If large purchase datasets from retailers were to become routinely available to outbreak investigators, comparisons could be made between case and non-case consumers. Thus, odds ratios for purchase could be calculated immediately and the process of searching for candidate foods (hypothesis generation) and the subsequent step of assessing their likelihood as outbreak vehicles (analytical epidemiology) could be performed in a single step. In addition, the methods may be a powerful tool for product identification, trace-back/trace-forward investigation and assessing likelihood of a food being an outbreak vehicle through comparisons of distribution and intensity of sales. A purchase data analysis could provide codes identifying the foods uniquely, such as European/International Article Numbering (EAN) or Global Trade Item Number (GTIN). This may potentially lead to efficient and fast comparative analyses using food databases. The latter is important, because trace-back investigations for larger outbreaks may reach levels of complexity where they become impossible to perform with traditional methods in addition to being lengthy and labour-intensive.

Such a framework would be strengthened by the increased penetration of card or mobile phone-based payments, expected to occur in the coming years. Combined with the foreseen increased application of whole-genome sequencing for routine surveillance of food-borne infections, it might also be valuable for the investigation of small or protracted outbreaks from continuous sources where cases are currently regarded as sporadic. Likewise, it may also be valuable for source attribution purposes, i.e. to describe the relative distribution of the sources which give rise to sporadic cases. Finally, as seen in one outbreak [7], it may be used to find and warn customers who have bought a product found to be contaminated and may thereby also help stop further cases [27].

Importantly, however, a number of requirements of a structural nature would need to be resolved before widespread use of the method could take place. These requirements include legal frameworks for ensuring consumer protection and patients’ privacy and the need to establish and maintain agreements between public health institutions and retailers securing data access. Data protection regulations and other obstacles for data access differ between countries and this may be the reason for why application of the method was geographically skewed. Adding to that, a number of more general methodological obstacles exist. First, purchase does not equal consumption and cases may often be part of families or households so that food purchases by several persons may need to be collected. Secondly, capturing foods consumed in restaurants or smaller retailers including convenience food remains a challenge, and thirdly, purchases made without the use of loyalty or payment cards will go unnoticed with current coverage and payments systems. Finally, not all retailers may wish to share data, affecting the coverage of the purchasing data. However, even if only imperfect data can be retrieved, the method may still produce results. An analogy can be drawn with standard disease surveillance, which often also captures only a fraction of all cases, but nonetheless is useful for finding and solving outbreaks. Hence, incompleteness in exposure assessment should not preclude efficient use of the method.

Overall, the papers we found and included contained little detail on how purchase data analysis was applied. The handling of data was most often not described in detail. With few exceptions [18,21], the total number of receipts retrieved, the period and the fraction of total purchases these receipts covered were not accounted for. Also, restrictions or obstacles of a legal, cultural or habitual nature were generally not mentioned and we could therefore not extract data on such matters. The papers did in general mention good working relationships between public health authorities and food retailers. Efforts to protect citizen privacy were not described in detail. Secure systems to handle potentially sensitive purchase data, systems to obtain consent, and share data are prerequisites of a wider implementation of consumer purchase datasets, and descriptions hereof in future studies would be beneficial.

This review has several limitations. A broader literature search including more search terms, languages other than English or including unpublished outbreak reports might have revealed more studies. We also limited our search to after the year 2005, but we note that studies taking advantage of shopping receipts in paper form also exist from before this time [28]. The papers generally report successful use of consumer purchase data; however, this could be partly due to publication bias, which is known to affect reporting of food-borne outbreaks [29]. We found one example where consumer purchase data were used for investigation of a large outbreak without finding the source [19], but it is possible that more unresolved and unpublished outbreaks using the consumer data method exist.

In conclusion, the reviewed papers describe a powerful outbreak investigation method. It holds promise of developing into a routinely applied tool provided that more automated procedures reducing labour for retailers as well as epidemiologists and ways of making data more available could be found. We envision a near future where food purchase information in some countries can be automatically collected from cases of food-borne infections and compared with that of a large panel of non-cases. Such a system would significantly improve source-identification and risk-assessment efforts, facilitate efficient trace back enabling timely interventions and reduce illness caused by food-borne pathogens.
